# Evaluation of the Flexural Behavior of One-Way Slabs by the Amount of Carbon Grid Manufactured by Adhesive Bonding

**DOI:** 10.3390/polym16192690

**Published:** 2024-09-24

**Authors:** Kyung-Min Kim, Sung-Woo Park, Bhum-Keun Song, Seon-Hee Yoon

**Affiliations:** 1 Seismic Safety Center, Korea Conformity Laboratories (KCL), Suwon 16229, Republic of Korea; 2 Korea Carbon Industry Promotion Agency, Jeonju 54853, Republic of Korea; 3 High In Tech Co., Ltd., Ulsan 44785, Republic of Korea

**Keywords:** carbon grid, CFRP reinforcement, flexural behavior, pultruded CFRP strands, textile-reinforced concrete

## Abstract

Fiber-reinforced polymers (FRPs), which are resistant to corrosion, are used as reinforcement material for concrete. However, the flexural behavior of concrete members reinforced with FRPs can vary depending on the properties of FRPs. In this study, the flexural behavior of one-way concrete slab specimens reinforced with a new grid-type carbon-fiber-reinforced polymer (CFRP) (carbon grid) manufactured by bonding pultruded CFRP strands to an adhesive was investigated. The experimental results indicated differences in the load–deflection relationships of the specimens depending on the carbon grid reinforcement amount. Specimens in which the carbon grids were over-reinforced or reinforced close to the balanced reinforcement ratio reached the maximum load due to concrete crushing and exhibited ductile failure. The specimen under-reinforced with the carbon grid exhibited brittle failure. Specimens with carbon grid reinforcement close to a balanced reinforcement ratio exhibited maximum loads ranging from 0.43 to 0.61 times the calculated flexural strength, which resulted in becoming 0.86–1.00 lower in the specimens with a wider width of the CFRP strands. This study proposes coefficients to estimate the stiffness of carbon-grid-reinforced concrete flexural members after cracking. Applying these coefficients resulted in stiffness calculations that reasonably simulated the behavior of the specimens reinforced with carbon grids after crack formation.

## 1. Introduction

The most common structural form since the 20th century has been reinforced concrete (RC). When exposed to marine environments, chloride ions can penetrate cracks on the surface, causing rebar corrosion and concrete damage, which can lead to rapid deterioration in the durability of structural members [1,2,3]. Chloride-induced damage in reinforced concrete structures is prevented through regulations on the minimum cover thickness during design and meticulous construction to minimize voids between the concrete and rebars. In addition, attempts have been made to prevent rebar corrosion by coating rebar surfaces with epoxy or other materials [4,5,6,7]. However, this procedure has been limited to a few bridges because of the localized corrosion caused by damage to the epoxy coating during concrete construction [6] and the decreased bond between the concrete and the epoxy-coated rebar [7].

Since the 1970s, efforts have been made to prevent fundamental chloride-induced damage in reinforced concrete structures caused by rebar corrosion by replacing rebars with corrosion-resistant fiber-reinforced polymer (FRP) [8,9]. Bar-type FRP, primarily with a circular cross-section, is produced using fibers such as glass fiber; carbon fiber; and aramid fiber; and thermosetting resins such as epoxy, vinyl ester, and so on, and is applied to bridge slabs [8,9,10]. Recently, grid-type FRPs, which can also be considered as a type of tensile reinforcement in textile-reinforced concrete (TRC) [11,12], have been developed [13,14,15,16,17,18,19,20,21,22,23,24,25,26,27,28,29] and are being applied in the construction of new structures such as building slabs and walls [25,26], bridge foundations [27], and in the reinforcement of existing structures [28,29]. 

FRP reinforcements have difficulties regarding bending in construction sites and recycling because they use thermosetting resins as matrix materials. On the other hand, thermoplastic resins are considered to be easier to reshape and recycle compared to thermosetting resins because they are thermosoftening polymers that can reshape at a specific temperature and harden when cooling [30]. Accordingly, as an alternative to the difficulties of bending at construction sites and recycling of FRP reinforcements, some bar-type fiber-reinforced thermoplastic polymer (FRTP) reinforcements have been developed using thermoplastic resins such as polyethylene (PE), polypropylene (PP), polyvinyl chloride (PVC), polystyrene (PS), etc., as matrix materials [31,32].

To prevent brittle failure in RC structures, members are typically designed such that failure is initiated by the yielding of the rebar when determining the flexural strength by design standards such as ACI 318-19 [33]. In contrast, concrete members reinforced with FRPs can be designed to achieve flexural strength through either FRP rupture or concrete crushing [8]. When designing members to achieve flexural strength by FRP rupture, the brittle failure characteristics of the fibers cause them to exhibit brittle failure behavior upon reaching the flexural strength [21,27]. Conversely, when designing members to achieve flexural strength by concrete crushing, the load is subsequently borne by the FRP reinforcement, and ductile behavior is observed over a certain range [34]. However, experimental research on the failure behavior of concrete members reinforced with FRPs is limited [35,36,37,38].

Grid-type FRP reinforcements form grids by arranging FRP strands with flat cross-sections in both warp and weft directions. Therefore, grid-type FRP reinforcements have advantages in terms of construction, not only for the construction of new structures but also for strengthening existing structures because there is no need for the assembly work of longitudinal and transverse reinforcements at construction sites. Additionally, grid-type FRP reinforcements are effective in reducing the cross-sectional thickness of members due to the generally thin thickness of FRP strands. Meanwhile, the cross-sectional shape, size, and spacing of the FRP strands differ from those of conventional rebars and bar-type FRP reinforcements. Bar-type FRP reinforcements generally have similar cross-sectional shapes and rebar sizes and are placed at similar spacing as rebars, too. Thus, the flexural behavior of concrete members reinforced with bar-type FRP reinforcements is known to be similar to that of RC members except for the greater decrease in stiffness after cracking [39]. However, the cross-sectional width and thickness of the FRP strands that constitute grid-type FRPs are smaller than those of the rebars, and the spacing between them is smaller than the typical spacing between the rebars. Consequently, the flexural behavior of concrete members reinforced with grid-type FRPs is characterized by multiple continuous cracks with temporary load reductions followed by load increases [15]. This results in a significant reduction in stiffness, and eventually, the member reaches flexural strength with grid-type FRP reinforcement, thereby resisting the load without the formation of new cracks.

Grid-type FRP reinforcements are generally formed by integrating the intersections of FRP strands arranged at regular intervals in warp and weft directions [17,24]. The major methods for grid formation include using separate long fibers for knitting in the warp direction to create a biaxial warp-knitting structure [17] and stacking multiple FRP strands in warp and weft directions, followed by resin curing, to form a cross-laminate structure [24]. 

Among the reinforcing fibers for FRP reinforcements, carbon fiber (CF) has exceptional features of lightweight, superior load-bearing strength at high temperatures, low density, high tensile strength and modulus, and excellent chemical resistance [30]. In this study, a new grid-type carbon fiber-reinforced polymer (CFRP) (carbon grid), KC, was developed as a reinforcement for concrete by manufacturing thin-flat CFRP strands using a pultrusion process and bonding them with an adhesive to form grids in both warp and weft directions. CF was used as a reinforcing fiber to ensure high tensile strength (over 2000 MPa) and modulus (over 150 GPa) of grid-type FRP reinforcement with small cross-sectional areas of FRP strands. The performance of carbon grid KC as a reinforcement for concrete was experimentally evaluated. Accordingly, one-way concrete slab specimens reinforced with carbon grid KC were prepared with experimental variables, including the effective depth, amount of carbon grid reinforcement, the presence of transverse CFRP strands, and the width of the CFRP strands. A four-point bending test was performed to evaluate the flexural behavior of the members reinforced with a carbon grid KC. In addition, the performance of carbon grid KC was compared with that of the rebars using four-point bending tests on the one-way slab specimens. 

## 2. Materials and Methods

### 2.1. Experimental Design

#### 2.1.1. Material Properties

**Figure 1:**
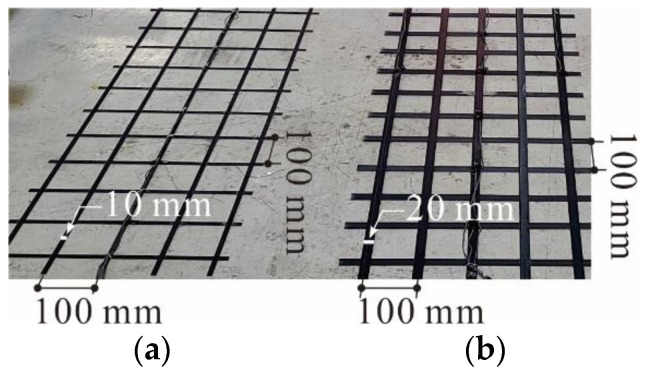
Carbon grids: (**a**) KC_w10; (**b**) KC_w20.

**Figure 2:**
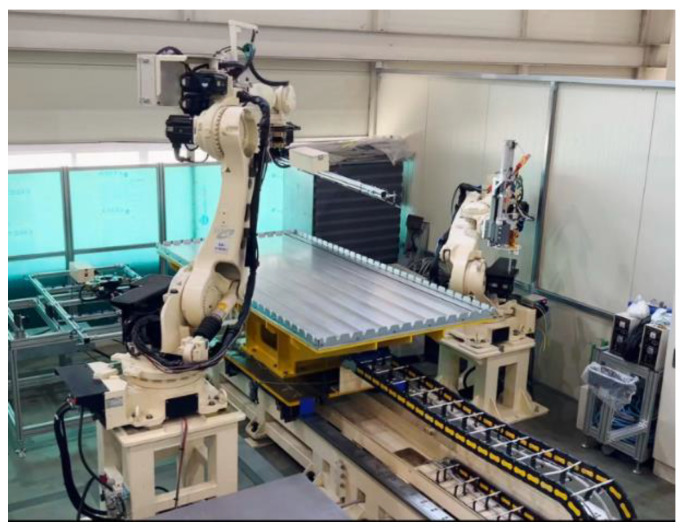
Manufacturing robots for carbon grid KC.

The carbon grid KC used in this study is shown in Figure 1. The carbon grid KC was manufactured by arranging CFRP strands made by impregnating carbon fibers (Hyosung, Republic of Korea, Jeonju, H2550 12K) with epoxy (Huntsman, The Woodlands, TX, USA, ARALDITE^®^ CY 5192-1) through a pultrusion process into thin-flat plates with a width of 20/10 mm and a thickness of 1 mm. These strands were placed horizontally at 100 mm intervals, with a high-strength rapid-curing adhesive of 20 MPa (Bostik, France, Colombes, SAF 30-15) applied at the intersections of the horizontal and vertical CFRP strands. Vertical CFRP strands were then arranged at 100 mm intervals and compressed at the intersections to complete the manufacturing process [34]. The use of strands manufactured through a pultrusion process is advantageous for securing the tensile strength in a carbon grid KC. In addition, the entire manufacturing process of the carbon grid KC, including the horizontal and vertical placement of the CFRP strands, adhesive application, and transportation of the completed carbon grid, was automated using robotic production equipment (High In Tech Co., Ltd., Ulsan, Republic of Korea), as presented in Figure 2. This automation ensures the precise and rapid production of grid-type FRP and allows for the manufacturing of grids using different materials and shapes of FRP strands.

Table 1 summarizes the characteristics of the carbon grid KC and rebar, as well as the tensile test results of the CFRP strands with the carbon grid KC and rebar. Tensile tests were conducted on the CFRP strands using ASTM D7205 [40] and CAN/CSA-S806-02 [41] as references. The tensile specimens were prepared for these tests by attaching steel plates to both ends of the CFRP strands and filling the spaces between the plates with epoxy. The specimens were then subjected to a loading rate of 5 mm/min using a 1000 kN universal testing machine. Meanwhile, rebars of d10 and d13 were used as rebars, and their tensile properties were tested based on ISO 6935 [42].

The tensile strength and modulus of elasticity of the CFRP strands in carbon grid KC_w20 were 97.6% and 84.9% of those of the CFRP strands in carbon grid KC_w10, respectively. This indicates that the CFRP strands in carbon grid KC_w10 have a higher tensile strength and modulus of elasticity. Compared with the rebars, the CFRP strands used in the carbon grid KC exhibited an average tensile strength that was 377.2% higher but only 82.1% of the tensile modulus of elasticity.

**Figure 3:**
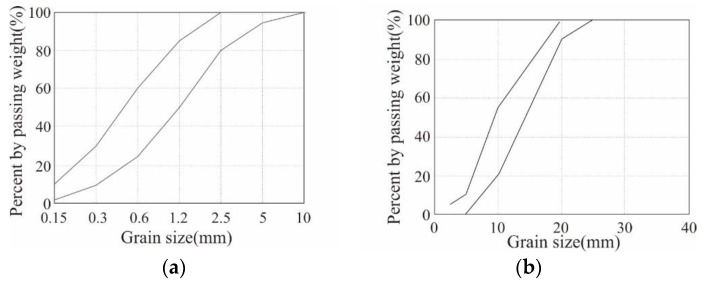
Standard particle size distribution curve: (**a**) find aggregate; (**b**) coarse aggregate.

Table 2 summarizes the mix design and 28-day compressive strength of the concrete. The concrete had a water/cement ratio of 35% and coarse aggregates with a maximum size of 25 mm were used. Coarse aggregates and fine aggregates were used according to the standard particle size distribution curve by KS F 2527 [43], as shown in Figure 3. According to the compression tests conducted following ISO 1920-4 [44], the 28-day compressive strength of the concrete was approximately 42.7 MPa.

#### 2.1.2. Specimen Overview

**Figure 4:**
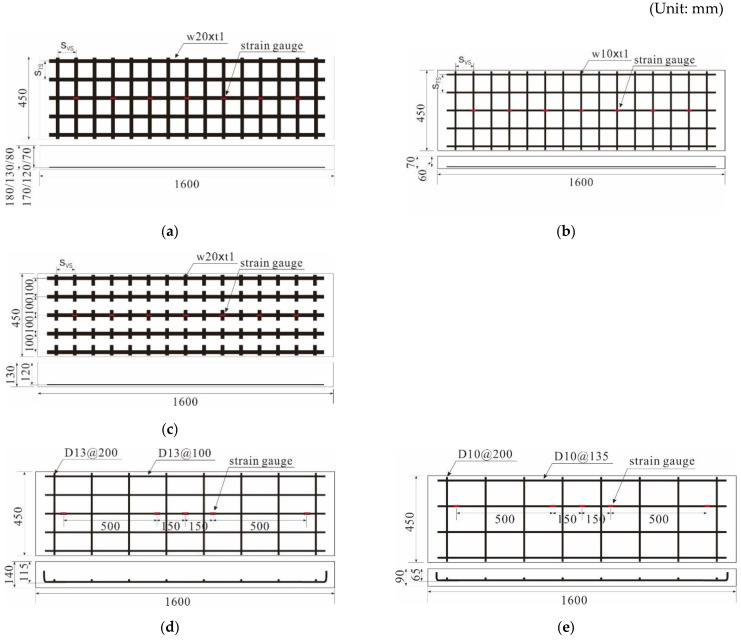
Specimens: (**a**) K200_d120_1, K200_d120_2, K200_d170, and K200_d70; (**b**) K200_d60; (**c**) K200_d120_s; (**d**) RC_d109; (**e**) RC_d60.

To evaluate the applicability of the carbon grid KC in concrete structures, one-way slab specimens of carbon-grid-reinforced concrete were prepared using two types of carbon grid KC, as shown in Figure 1 (also see Table 3 and Figure 4). The main experimental variables were the effective depth of the specimens, the corresponding amount of carbon grid reinforcement, the presence of CFRP strands in the transverse directions, and the width of the CFRP strands. These experimental variables are the main design factors for flexural concrete members reinforced with any type of reinforcement such as rebars, bar-type FRP reinforcements, and grid-type FRP reinforcements. Additionally, for comparison with conventional tensile reinforcements, one-way concrete slab specimens reinforced with rebars were prepared. The reinforcement ratios of carbon-grid-reinforced specimens were 0.13–0.32, and those of RC specimens were 0.70–1.10. This is because the carbon grid KC had a tensile strength of more than 3.5 times that of rebars and was manufactured with smaller cross-sectional areas compared with rebars. Unlike RC structures, members reinforced with FRP strands do not require a minimum cover thickness to prevent chloride-induced damage owing to their chemical stability [45]. Therefore, the cover thickness of the carbon-grid-reinforced specimens was set to 10 mm.

**Figure 5:**
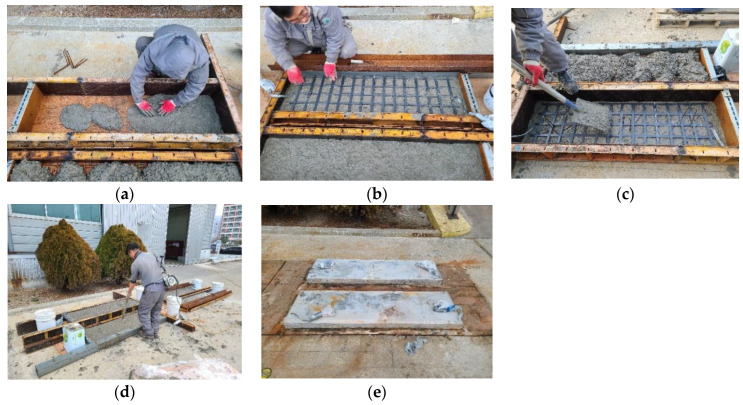
Manufacturing of specimens reinforced with carbon grids: (**a**) 1st concrete pouring; (**b**) arrangement of carbon grid; (**c**) 2nd concrete pouring; (**d**) vibrating; (**e**) form removal and curing.

Carbon-grid-reinforced specimens were fabricated as shown in Figure 5. Initially, concrete was poured to a height of 10 mm at the bottom of the formwork in order to install the carbon grid at a distance equal to the cover thickness from the bottom of the formwork, as shown in Figure 5a. Subsequently, the carbon grid was placed (Figure 5b), and additional concrete was poured into the full height of the specimen, as shown in Figure 5c. As shown in Figure 5d, vibration compaction was also performed during concrete pouring. Finally, the formwork was removed one day after pouring, and the specimens were cured for at least 28 d in an outdoor environment, as shown in Figure 5e. Meanwhile, spacers can be used to install the carbon grids at specific locations within the formwork during concrete pouring on a construction site [46], but the specimens were manufactured without spacers considering the specimen size and influence of spacers, etc., in this study.

Table 4 summarizes the crack strength and flexural strength of the specimens, calculated based on the results of the material tests presented in Table 2 and Table 3 with the assumption of the equilibrium of forces and strain compatibility.

Among the carbon-grid-reinforced specimens, only KC200_d70 had a carbon grid reinforcement that exceeded the balanced reinforcement ratio (over-reinforced), indicating that compressive failure due to concrete crushing at the compression edge was expected to precede the rupture of the carbon grid reinforcement. For all the other specimens that had carbon grid reinforcement below the balanced reinforcement ratio (under-reinforced), failure was expected to occur from the rupture of the carbon grid reinforcement. The RC specimens RC_d109 and RC_d69 were used for comparison with the flexural behavior of the carbon-grid-reinforced specimens at effective depths of 120 mm and 170 mm, respectively. Both were designed to undergo final failure by the yielding of the rebars.

### 2.2. Loading and Measurement Method

**Figure 6:**
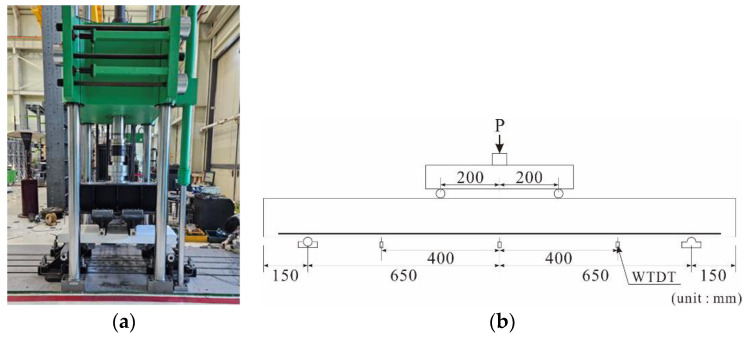
Test setup: (**a**) universal test machine; (**b**) loading and measurements.

A four-point bending test was performed with a 1000 kN dynamic fatigue universal test machine (UTM) (Figure 6a), and a load was applied to points 200 mm away from the center on either side of the specimens with a displacement control of 5 mm/min.

The loads applied to the specimens were measured using a load cell integrated into the UTM. The deflection of the specimens was measured using a total of six wire-type displacement transducers (WTDTs) (Tokyo Measuring Instruments Lab, Tokyo, Japan), which were installed at three locations on the underside of the specimens, with two WTDTs at each location, as illustrated in Figure 6b. The deformation occurring in the CFRP strands of the carbon grids was measured using strain gauges [32]. Accordingly, strain gauges were attached to the surfaces of the CFRP strands and rebars to measure their deformation. Seven strain gauges were attached to the surface of the CFRP strands in the tensile axial direction at the center of the cross-section of the carbon-grid-reinforced specimens, as illustrated in Figure 4a–c. For specimen RC_d109, five strain gauges were attached to the surface of the main rebar located at the center of the specimen cross-section, and for specimen RC_d60, five strain gauges were attached to the surface of one of the two main rebars located at the center of the specimen cross-section, as illustrated in Figure 4d,e, respectively.

## 3. Results and Discussion

### 3.1. Crack and Failure Geometry

**Figure 7:**
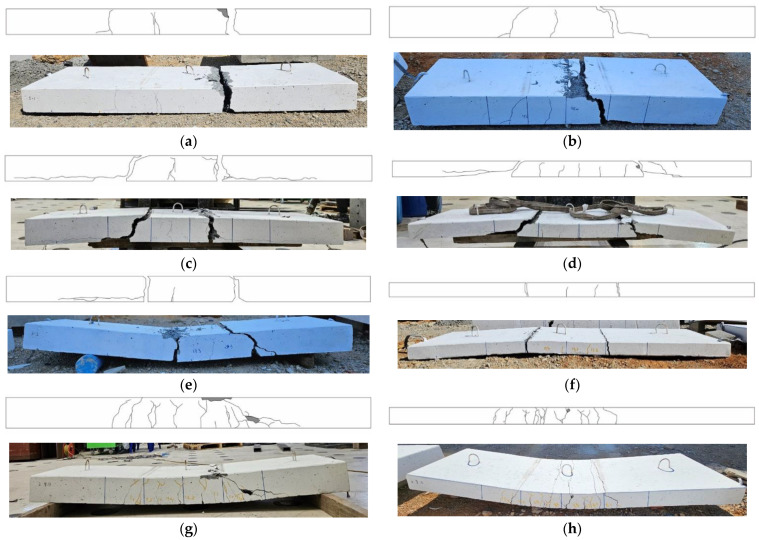
Failure of the specimens: (**a**) K200_d120_1; (**b**) K200_d170; (**c**) K200_d120_2; (**d**) K200_d70; (**e**) K200_d120_s; (**f**) K200_d60; (**g**) RC_d109; (**h**) RC_d60.

Table 5 summarizes the number of cracks and the average crack space in the specimens, and Figure 7 presents the final failure geometries of the specimens. The number and spacing of cracks have a relationship with the bond between concrete and the carbon grid, and stress concentration due to low bonding results in fewer cracks. Here, the number and spacing of flexural cracks on the surface of the specimens were measured at the location of the carbon grids.

Compared to the RC specimens, the carbon-grid-reinforced specimens exhibited fewer cracks and wider crack spacing. Except for specimen K100_d60, the carbon-grid-reinforced specimens developed bonding cracks at the carbon grid locations, with concrete spalling observed beneath the carbon grid, indicating a poorer bond between the carbon grid and concrete compared to the RC specimens. Ultimately, the carbon-grid-reinforced specimens experienced the widening of one or two cracks that penetrated the specimen cross-section, leading to the slippage of the carbon grid between the widened cracks. This resulted in the separation of the specimens into two or three segments connected by a carbon grid.

Except for specimen K200_d70, which was over-reinforced with a carbon grid, the carbon-grid-reinforced specimens developed cracking primarily in the flexural region with minimal crack distribution in the flexural-shear region. The crack spacing ranged from 147 to 219 mm, which was wider than the spacing of the longitudinal CFRP strands of the carbon grid. In addition, greater effective depths in the specimens correspond to wider crack spacing. In contrast, the over-reinforced specimen K200_d70 exhibited a significant number of flexural-shear cracks, similar to those in the RC specimens. The crack spacing was the narrowest among the carbon-grid-reinforced specimens, measuring 101 mm, which was comparable to the spacing of the longitudinal CFRP strands in the carbon grid. 

**Figure 8:**
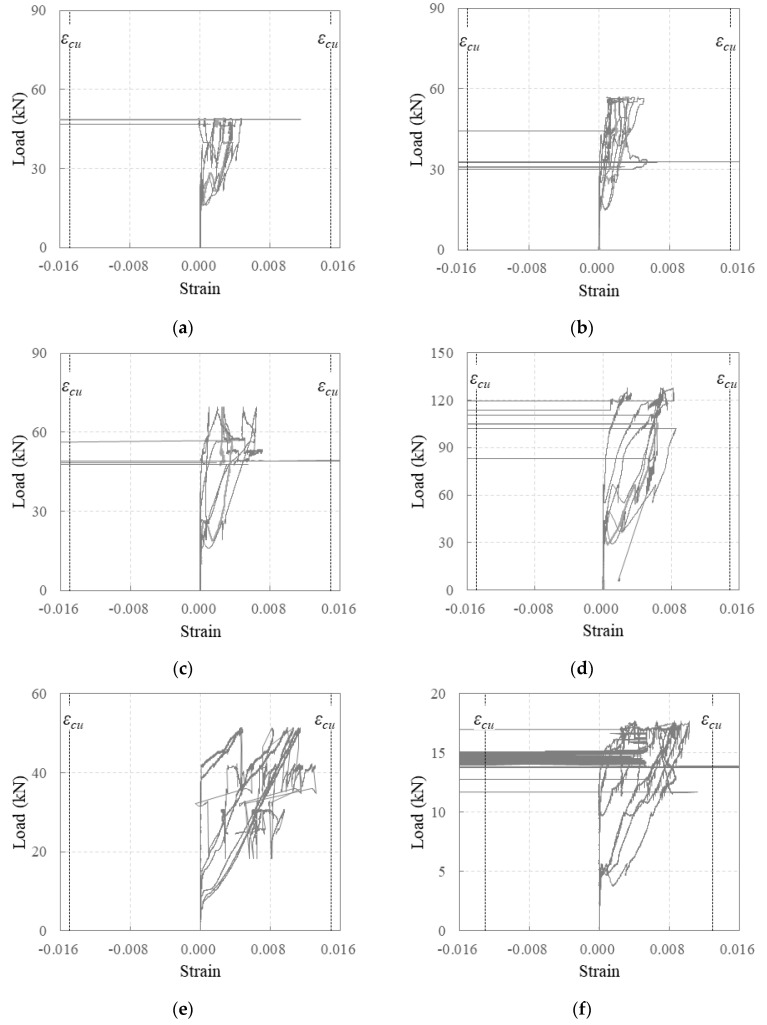
Relationship between the load and strain of specimens: (**a**) K200_d120_1; (**b**) K200_d120_; (**c**) K200_d120_s; (**d**) K200_d170; (**e**) K200_d70; (**f**) K200_d60.

Figure 8 shows the relationship between the load and strain of the carbon-grid-reinforced specimens. *ε_cu_* in Figure 8 denotes the strain at the tensile strength. The strain was measured using seven strain gauges attached to the longitudinal CFRP strands of the carbon grid, as shown in Figure 3. Significant fluctuations were also observed in the strain variation, which are believed to be caused not only by concrete damage, such as cracks, and tensile deformations of the strands but also by damage at the interface between the CFs and resin on the surface of the strands. 

**Figure 9:**
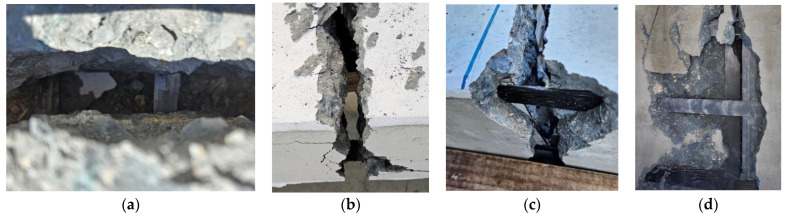
Damage observed in the carbon grids of specimens: (**a**) KC200_d120_1; (**b**) KC200_d120_2; (**c**) KC200_d120_s; (**d**) KC200_d170.

In the specimens that were under-reinforced with carbon grids, the tensile strains exceeded the strain at the tensile strength of the longitudinal CFRP strands of the carbon grids. All the longitudinal CFRP strands in the specimens experienced tensile strains beyond the strain at the tensile strength; however, only partial fiber rupture occurred in the longitudinal CFRP strands of specimen K200_d170, which had the greatest effective depth, as shown in Figure 9d. No full cross-sectional rupture of the longitudinal CFRP strands was observed. For specimen K200_d120_i with equal effective depths and amounts of carbon grid reinforcement, only interfacial failure between the fibers and resin occurred in the longitudinal CFRP strands, as shown in Figure 9a–c. 

### 3.2. Load–Deflection Relationship

Table 6 and Table 7 summarize the load, mid-span deflection, and stiffness at each stage of the specimens. In concrete members reinforced with carbon grids, the flexural behavior can be categorized into stages before crack formation, crack formation, and crack stabilization [21].

The crack stabilization stage refers to the phase in which no further cracking occurs as the load increases, spanning from the point of the last crack formation to the maximum load point. The load and deflection at the “crack stabilization point” in Table 6 denote the load and deflection at the beginning of the crack stabilization stage, where the cracks last. Furthermore, the stiffness at each stage in Table 7 represents the slope of the line connecting the origin and crack formation points, crack formation and crack stabilization points, and crack stabilization and maximum load points.

For specimen K200_d120_i with equal effective depths and amounts of carbon grid reinforcement, the load, mid-span deflection, and stiffness at the crack formation stage were not significantly different. However, specimen K200_d120_s, in which the CFRP strands were not arranged in the transverse direction, exhibited a smaller load and mid-span deflection at the crack stabilization point than specimens K200_d120_1 and K200_d120_2, which developed cracks simultaneously. However, the stiffness increased during the crack stabilization stage, leading to a higher load and mid-span deflection at the maximum load point. This suggests that the specimens in this study were one-way, with the load affecting only the longitudinal CFRP strands. This indicates that the transverse CFRP strands did not significantly influence the strength of the specimens. 

However, specimens KC200_70 and KC100_60, with smaller effective depths, exhibited lower stiffness both before and after crack formation than the other specimens. They also exhibited larger deflections at the maximum load point.

**Figure 10:**
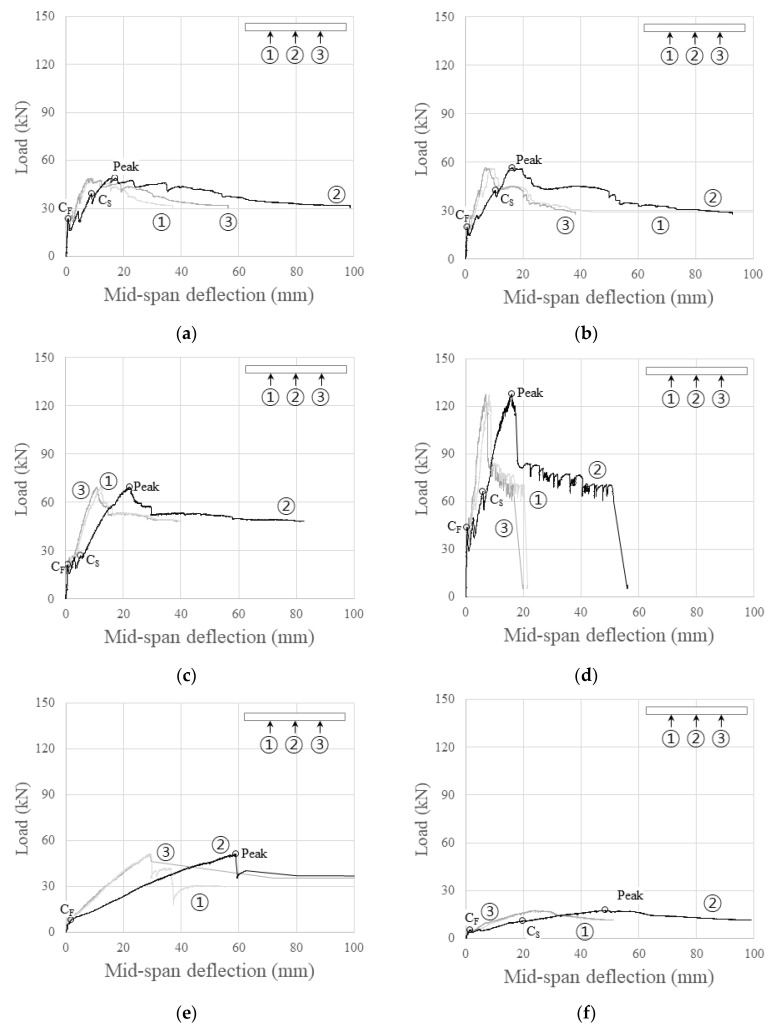
Relationship between load and mid-span deflection for specimens: (**a**) K200_d120_1; (**b**) K200_d120_2; (**c**) K200_d120_s; (**d**) K200_d170; (**e**) K200_d70; (**f**) K200_d60.

Figure 10 depicts the load–deflection relationship of the specimens, with the numbers (①, ②, and ③) indicating the positions of the three WTDTs installed, as illustrated in Figure 5, representing the relationship between load and deflection measured at each WTDT location.

All specimens exhibited the largest deflection at the mid-span under the same load. While some specimens, such as K200_120_1, experienced increasing deflection on the right side, where the load was concentrated after the maximum load point, most specimens showed similar deflections on the left and right sides.

Among the specimens under-reinforced with carbon grids, the load after the peak decreased to 63.5% of the maximum load for K200_170. As the load was transferred through the carbon grid, it initially exhibited ductile behavior until an abrupt load drop led to brittle failure. This was attributed to failure at the interface between the carbon fibers and the resin of some CFRP strands in specimen K200_170, as shown in Figure 9d, where the carbon fibers experienced fracture. In contrast, the under-reinforced specimens, except for K200_d170, exhibited a decrease in load after the maximum load point. However, their behavior was relatively ductile until the end of the test, gradually decreasing in load, as shown in the observed in Figure 9a–c. While the interface between the carbon fibers and the resin of the CFRP strands in these specimens was damaged, the carbon fibers did not fracture completely, allowing the longitudinal CFRP strands to resist the load.

Specimen K200_d70, which was over-reinforced with carbon grids, exhibited characteristics different from those of other carbon-grid-reinforced specimens, in that it did not exhibit a pattern of load decrease and increase in response to crack formation. It exhibited a gradual deterioration in the stiffness of the load–deflection relationship as it approached the peak due to concrete crushing. In addition, after reaching the peak, the load decreased as it was transferred through the carbon grid. The strains in the longitudinal CFRP strands were smaller than the strain at tensile strength, and there was no damage arising from interface failure between the carbon fibers and resin; that is, ductile behavior was exhibited until the end of the test, with no significant decrease in load despite greater deformation.

**Figure 11:**
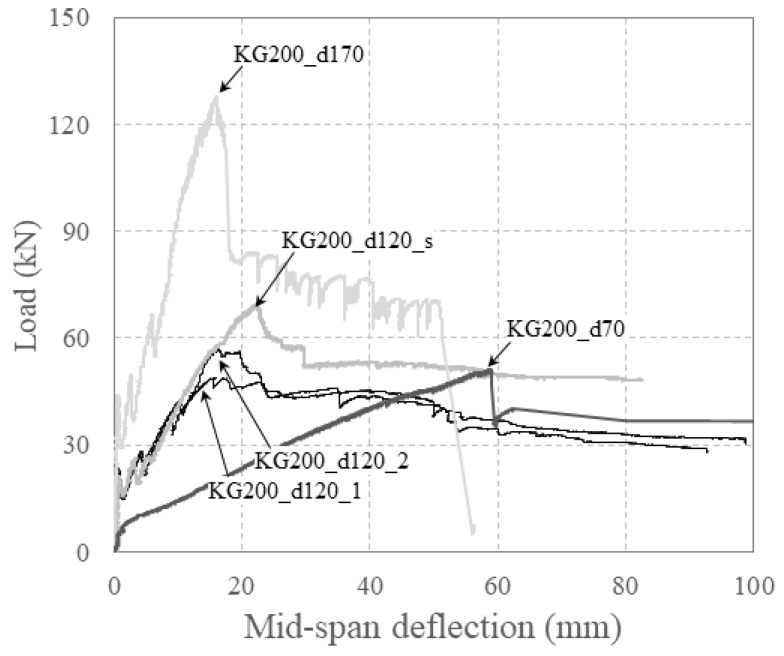
Relationship between load and mid-span deflection for specimens considering effective section depth.

Figure 11 illustrates the relationship between the load and mid-span deflection with respect to effective depth for specimens reinforced with carbon grid KC_w20.

As the effective depth increased, the moment of inertia also increased, resulting in an increase in the stiffness of the specimen, particularly after crack formation. As the effective depth increased, the specimens reached their peaks at smaller deflections.

Moreover, the maximum load of the specimens tended to increase with effective depth. However, specimens KG200_d120_1 and KG200_d120_2 had larger effective depths compared to KG200_d70, which were expected to result in a flexural strength that was 2.24 times larger, as summarized in Table 4. Contrary to expectations, the experimental results indicated that the maximum loads for specimens KG200_d120_1 and KG200_d120_2 were only approximately 0.95–1.15 times that of specimen KG200_70. Specimen KG200_d120_1 exhibited a lower maximum load. This discrepancy can be attributed to concrete crushing at the right loading point in specimen KG200_d120_1, which concentrated the load and caused significant crack widening, preventing a substantial increase in the load.

### 3.3. Flexural Behavior Evaluation 

#### 3.3.1. Crack and Maximum Load

**Figure 12:**
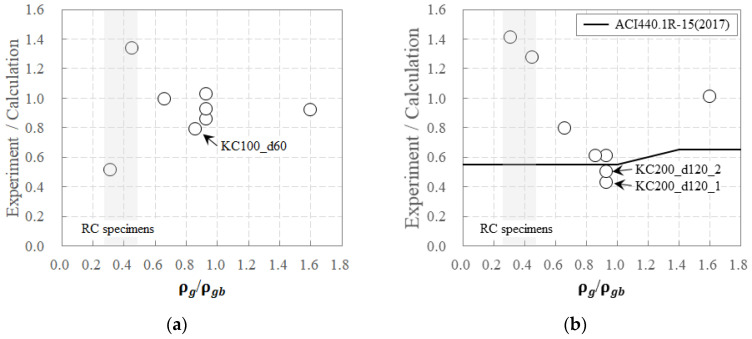
The strength ratio of experimental and calculation results according to $\rho_{g}/\rho_{gb}$: (**a**) crack; and (**b**) maximum loads.

Figure 12 compares the ratio of experimental to calculated values for crack and maximum loads based on $\rho_{g}/\rho_{gb}$.

For the RC specimens, there was a significant variation in the ratio of the experimental to calculated crack load values across different specimens. However, for the carbon-grid-reinforced specimens, this ratio exhibited little variation among the specimens. Among the specimens reinforced with carbon grid KC_w20, the experimentally measured crack loads were between 86% and 103% of the calculated crack loads, as listed in Table 4. As $\rho_{g}/\rho_{gb}$ increased, indicating a higher amount of carbon grid reinforcement in the cross-section, the ratio of experimental to calculated crack load values generally decreased. However, for the specimen reinforced with carbon grid KC_w10, the ratio of the experimental to the calculated crack load value was 0.79, which was the lowest among the carbon-grid-reinforced specimens.

For the maximum load, the RC specimens had experimental values that were 127–141% higher than the calculated values. In contrast, for the carbon-grid-reinforced specimens, only the over-reinforced specimen KG200_d70 exhibited an experimental maximum load similar to the calculated value, with a ratio of 101%. The other carbon-grid-reinforced specimens exhibited experimental values that were lower than the calculated values. This discrepancy is attributed to insufficient bonding between the carbon grid and concrete, leading to premature concrete crushing at lower load levels before the longitudinal CFRP strands reached their tensile strength and load concentration at one of the two loading points, as presented in Figure 6.

According to ACI 440.1R-15 [8], to ensure the safety of flexural members reinforced with FRPs, a strength reduction factor of 0.55–0.65 should be applied, depending on the reinforcement condition and failure mode. In this study, the experimental results for the under-reinforced specimen KC200_d170 and the over-reinforced specimen KC200_d70 showed that the ratios of the experimental to calculated maximum loads were 0.79 and 1.01, respectively, both higher than the strength reduction factors recommended by ACI 440.1R-15 [8]. Conversely, for specimens KC200_d120_i and KC100_d60 with carbon grid reinforcement close to the balanced reinforcement ratio, the ratios of experimental to calculated maximum loads were in the range of 0.43–0.61 and 0.61, respectively, which are similar to or lower than the strength reduction factors suggested by ACI 440.1R-15 [8]. The specimens reinforced with the wider width of the CFRP strands exhibited 0.86–1.00 lower ratios of experimental to calculated maximum loads. Specifically, for specimens KC200_d120_1 and KC200_d120_2, which had CFRP strands arranged both longitudinally and transversely, the ratios of the experimental to calculated maximum loads were 0.43 and 0.50, respectively, representing 78% and 91% of the strength reduction factors specified in ACI 440.1R-15 [8].

#### 3.3.2. Stiffness of Cracked Section

The initial stiffness *K_E_* and post-cracking stiffness *K_cr_* of the carbon-grid-reinforced specimens, which were similar to those of the RC members, were evaluated using Equations (1) and (2) as follows [47,48]:

(1) $$K_{E}=\frac{48E_{c}I_{g}}{L^{3}}$$

(2) $$K_{cr}=\frac{48E_{c}I_{e}}{L^{3}}$$

where *E_c_* is the modulus of elasticity of the concrete (MPa), *I_g_* is the gross moment of inertia (mm^4^), *I_e_* is the effective moment of inertia (mm^4^), and *L* is the specimen span length (mm).

One-way slabs reinforced with FRPs are known to exhibit lower post-cracking stiffness than RC members because of the weak bond between the FRP reinforcements and concrete, making it difficult to expect the tension-stiffening effect of concrete [8,49]. Therefore, in ACI 440.1R-15 [8] and Bischoff and Scanlon [49], the concept of a weighted average of flexibility and a weighted average of the uncracked and cracked member stiffness, respectively, was proposed for members reinforced with FRPs. They presented Equations (3a) and (3b) for the effective moments of inertia.

(3a) $$I_{e}=\frac{I_{cr}}{1-\gamma {\left(\frac{M_{cr}}{M_{a}}\right)}^{2}[1-\frac{I_{cr}}{I_{g}}]}$$

(3b) $$I_{e}={\left(\frac{M_{cr}}{M_{a}}\right)}^{3}\beta_{d}I_{g}+(1-{\left(\frac{M_{cr}}{M_{a}}\right)}^{3})I_{cr}$$

where *I_cr_* is the cracked transformed moment of inertia (mm^4^), *M_cr_* is the cracking moment (Nmm), and *M_a_* is the applied service load moment (Nmm).

Based on the experimental and analytical results, Equations (3a) and (3b) were used to evaluate the stiffness reduction resulting from FRP reinforcement application, defined as γ in Equation (4) and $\beta_{d}$ in Equation (5).

(4) $$\gamma =1.72-0.72(M_{cr}/M_{a})$$

(5) $$\beta_{d}=0.2\frac{\rho_{g}}{\rho_{gb}}\leq I_{g}$$

where $\rho_{g}$ is the carbon grid reinforcement ratio, and $\rho_{gb}$ is the carbon gird reinforcement ratio producing balanced strain conditions.

**Figure 13:**
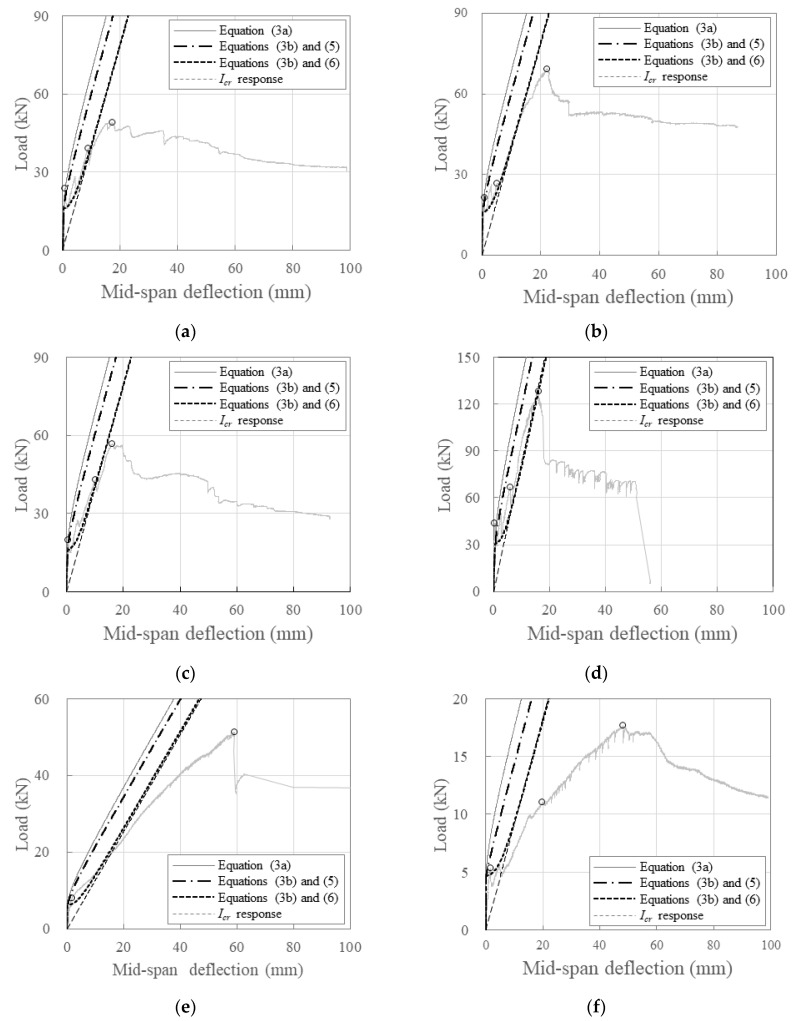
Comparison between the flexural behavior of specimens: (**a**) K200_d120_1; (**b**) K200_d120_2; (**c**) K200_d120_s; (**d**) K200_d170; (**e**) K200_d70; (**f**) K200_d60.

Figure 13 illustrates the relationship between the load and mid-span deflection using the flexural stiffness from Equations (1) and (2) along with the experimental results. The effective moments of inertia in Equation (2) were calculated using Equations (3a) and (3b) for comparison.

When calculating the post-cracking stiffness (*K_cr_*) using the effective moments of inertia from Equations (3a) and (3b), the load was significantly overestimated compared with the experimental results at the same deflection. This indicated an overestimation of the stiffness based on the experimental data using Equation (3a), which was more than when using Equation (3b). This discrepancy may occur because Equations (3a) and (3b) were derived from limited experimental studies primarily focusing on bar-type FRP reinforcements with circular cross-sections similar to rebars, particularly studies on glass-fiber-reinforced polymer (GFRP) reinforcements.

To achieve a more accurate simulation of the load–deflection relationship based on the experimental results, we calculated *I_e_* based on the stiffness of the crack formation stage from the crack formation point to the crack stabilization point, as summarized in Table 7. Considering this value and the ratio of the cracking moment to the crack stabilization moment, comparisons were made with the average *I_e_* calculated using Equation (3b). The experimental $\beta_{d}$ values were 0.03–0.08 times the values calculated using Equation (5), with the average being 0.05. Therefore, Equation (5) was modified to reflect the experimental results showing that $\beta_{d}$ was approximately 0.05 times lower on average, leading to Equation (6).

(6) $$\beta_{d}=0.01\frac{\rho_{g}}{\rho_{gb}}\leq I_{g}$$

As depicted in Figure 13, the post-cracking stiffness *K_cr_* modeled using Equations (6) and (3b) closely simulated the behavior of the carbon-grid-reinforced specimens after crack formation. This model was particularly accurate in replicating the behavior between the crack formation and crack stabilization points for specimens with larger effective depths.

### 3.4. Comparison of Flexural Behaviors with RC

**Figure 14:**
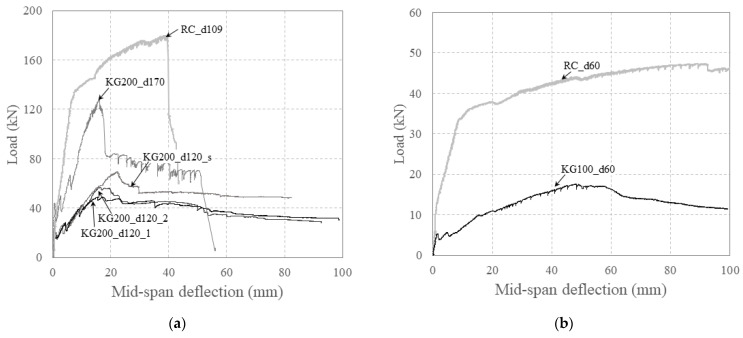
Flexural behavior of specimens reinforced with carbon grid and rebar: (**a**) larger effective depth; (**b**) smaller effective depth.

Figure 14 compares the relationship between the load and mid-span deflection of the carbon-grid-reinforced specimens and RC specimens.

The carbon-grid-reinforced specimens exhibited a significant reduction in stiffness after crack formation compared with the RC specimens. Among the specimens with larger effective depths, RC_d109 exhibited brittle failure owing to the propagation of a shear crack on the right side (see Figure 7g). However, among the carbon-grid-reinforced specimens, only KG200_d170 exhibited brittle failure owing to the rupture of some fibers in the longitudinal CFRP strands. In contrast, KG200_d120 exhibited ductile behavior until the end of the test, with the carbon grid resisting the load after the peak. Despite being designed with a flexural strength 1.14 times higher than that of specimen RC_d109, specimen KG200_d170 exhibited a maximum load of only 0.71 times that of RC_d109. Although specimens KG200_d120_i were designed with a flexural strength that was 0.80 times that of RC_d109, the test results exhibited that the maximum load of specimens KG200_d120_i was only 0.27–0.39 times that of RC_d109. Unlike the RC specimens, the carbon-grid-reinforced specimens did not achieve adequate bonding between the carbon grid and concrete, reaching their peaks because of concrete crushing at load levels lower than the designed flexural strength. Consequently, the maximum loads of the carbon-grid-reinforced specimens were significantly lower than those of the RC specimens.

Among the specimens with smaller effective depths, KC100_d60 and RC_d60, which had the same effective depth, reached their peaks following a significant reduction in stiffness upon crack formation and rebar yielding, respectively. Ductile behavior, characterized by a gradual decrease in the load, was also observed after the peaks. Similar to the other carbon-grid-reinforced specimens, specimen KC100_d60 exhibited weaker bonding between the carbon grid and concrete despite being designed with a flexural strength of 0.86 times that of specimen RC_d60. Consequently, the maximum load of specimen KC100_d60 was only 0.37 times that of specimen RC_d60.

## 4. Conclusions

This study evaluated the potential application of carbon grid KC in concrete structures by assessing the flexural behaviors of one-way concrete slab specimens reinforced with two types of carbon grid KC through a four-point bending test. The experimental variables included the effective depth, the amount of carbon grid reinforcement, the presence of transverse CFRP strands, the width of the CFRP strands, and the type of tensile reinforcement used in the concrete. The findings of this study can be summarized as follows:

1. The carbon-grid-reinforced specimens exhibited wide crack spaces and fewer cracks. Poor bond properties between the carbon grid and concrete, such as bonding cracks, were also observed on the surfaces. Ultimately, the specimens exhibited an increased crack width that penetrated the cross-section, leading to the slipping of the carbon grid and the separation of the specimens along the cracks. Despite the strain measurements indicating tensile strains exceeding those at the tensile strength in the longitudinal CFRP strands, the CFRP strand sections did not fracture. Instead, damage was observed in the form of interfacial failure between the fibers and resin, along with fiber rupture.
2. The relationship between load and mid-span deflection varied with the amount of carbon grid reinforcement used. The specimen over-reinforced or reinforced close to the balanced reinforcement ratio exhibited ductile failure, but the specimen under-reinforced with the carbon grid exhibited brittle failure. In particular, for specimens with carbon grid reinforcement close to the balanced reinforcement ratio, the ratios of the experimental to the calculated maximum loads were found to be 0.43–0.61, which were similar to or lower than the strength reduction factor of 0.6 suggested by the design standard. This indicates that achieving the expected flexural strength using carbon grids is challenging.
3. As the effective depth increased, the moment of inertia of the specimens also increased, resulting in a higher stiffness. This resulted in the specimens reaching maximum loads with smaller deflections. Because the experiment focused on one-way slab specimens, the transverse CFRP strands did not significantly influence the strength of the specimens. The specimens reinforced with a wider width of the CFRP strands also exhibited the 0.86~1.00 lower ratios of experimental to calculated maximum loads compared to the specimen reinforced with a narrower width of the CFRP strands.
4. Based on the experimental results, this study proposes a formula to assess the impact of poor bonds between the carbon grid and concrete on the post-cracking stiffness degradation of carbon-grid-reinforced concrete members. The post-cracking stiffness calculated using the proposed formula closely matched the experimental results.
5. Compared to the RC specimens, the carbon-grid-reinforced specimens exhibited a lower flexural strength, but they demonstrated ductile behavior regardless of the effective depth except for the specimens under-reinforced with carbon grids. The flexural behavior of one-way concrete slabs reinforced with carbon grids composed of thin-plate-shaped CFRP strands was still evaluated through a limited set of experiments. Therefore, further research is required to predict the flexural behavior of carbon-grid-reinforced concrete members, which is different from RC members.

## Figures and Tables

**Table 1:** Characteristics and tensile properties of reinforcements.

Name	Strand			Grid Geometry (Longitudinal (G__VS_) × Transverse (G__TS_) Spacing, mm)	Tensile Strength (MPa)	Tensile Modulus of Elasticity (MPa)
	Width (mm)	Thickness (mm)	Area (mm^2^)			
KC_w20	20	1	20	100 × 100	2271	151,994
KC_w10	10	1	10	100 × 100	2327	179,000
d13	-	-	126.7	-	637 (501)	186,516
d10	-	-	71.3	-	582 (469)	216,657

( ) Yield strength.

**Table 2:** Mix design and compressive strength of concrete.

Cement (kg/m^3^)	Water (kg/m^3^)	W/C (%)	Fine Aggregate (kg/m^3^)	Coarse Aggregate (kg/m^3^)	Compressive Strength (MPa)
455	159	35	765	900	42.7

**Table 3:** Specifications of the one-way slab specimens.

Specimen	Width (mm)	Depth/ Effective Depth (mm)	Length (mm)	Reinforcement	Reinforcement Ratio (%)
K200_d120_1	450	130/120	1600	KC_w20	0.19
K200_d120_2					
K200_d120_s					
K200_d170		180/170			0.13
K200_d70		80/70			0.32
K100_d60		70/60		KC_w10	0.19
RC_d109		140/109		d13@200 × d13@100	1.01
RC_d60		90/60		d10@200 × d10@135	0.70

**Table 4:** Crack and flexural strength results for the specimens.

Specimen	$\rho_{g}/\rho_{gb}$	Crack Strength		Flexural Strength		Failure Mode
		Moment (kNm)	Load (kN)	Moment (kNm)	Load (kN)	
K200_d120_i for i = 1, 2, s	0.93	5175	23.0	25,549	113.6	CFRP strand rupture
K200_d170	0.66	9943	44.2	36,195	160.9	CFRP strand rupture
K200_d70	1.60	1950	8.7	11,420	50.8	Concrete crushing
K100_d60	0.86	1512	6.7	6492	28.9	CFRP strand rupture
RC_d109	0.45	6049	26.9	31,787	141.3	Rebar yield
RC_d60	0.31	2500	11.1	7564	33.6	Rebar yield

$\rho_{g}$: carbon grid reinforcement ratio, $\rho_{gb}$: carbon grid reinforcement ratio producing balanced strain conditions.

**Table 5:** Crack formation of the specimens.

Specimen	Number of Cracks	Average Crack Space (mm)
K200_d120_1	4	176
K200_d120_2	3	200
K200_d120_s	3	191
K200_d170	3	219
K200_d70	8	101
K100_d60	4	147
RC_d109	10	67
RC_d60	10	74

**Table 6:** Load and deflection of mid-span results.

Specimen	Crack Formation Point (CF)		Crack Stabilization Point (CS)		Maximum Load Point		(1)/(3)	(2)/(3)
	Load ^(1)^ (kN)	Deflection of Mid-Span (mm)	Load ^(2)^ (kN)	Deflection of Mid-Span (mm)	Load ^(3)^ (kN)	Deflection of Mid-Span (mm)		
K200_d120_1	23.58	0.86	39.05	9.00	48.97	17.20	0.48	0.80
K200_d120_2	19.72	0.67	42.85	10.37	56.80	16.17	0.35	0.75
K200_d120_s	21.30	0.90	26.84	5.28	69.37	22.12	0.31	0.39
K200_d170	43.92	0.47	66.63	5.95	127.81	16.07	0.34	0.52
K200_d70	7.99	1.85	-	-	51.33	59.03	0.16	-
K100_d60	5.32	1.59	11.05	19.89	17.66	48.33	0.30	0.63
RC_d109	35.92	1.20	113.48	5.46	179.91	38.64	0.20	0.63
RC_d60	5.70	0.60	27.23	6.27	47.36	88.97	0.12	0.57

For the RC specimens, the crack stabilization state represents the steel-yielding state. The numbers (1), (2), and (3) are used to represent division equations for the two columns on the right of the table.

**Table 7:** Stiffness results.

Specimen	Stiffness (N/mm)		
	Before Crack Formation (Stage 1)	Crack Formation (Stage 2)	Crack Stabilization (Stage 3)
K200_d120_1	27,453	1900	1209
K200_d120_2	29,614	2383	2406
K200_d120_s	23,609	1265	2526
K200_d170	92,933	4147	6048
K200_d70	4326	-	758
K100_d60	3345	313	232
RC_d109	29,868	18,235	2002
RC_d60	9482	3795	244

For the RC specimens, the crack stabilization state represents the steel-yielding state.

## Data Availability

The original contributions presented in the study are included in the article, further inquiries can be directed to the corresponding author.
